# COVID-19 and Interacting Public Health Threats in Europe During 2020–2025: A Narrative Review

**DOI:** 10.3390/epidemiologia7030078

**Published:** 2026-06-02

**Authors:** Christos Ntais, Ioanna P. Chatziprodromidou

**Affiliations:** 1 Epidemiology Program, School of Science and Technology, Hellenic Open University, 26335 Patras, Greece; 2 Department of Public Health, Medical School, University of Patras, 26504 Patras, Greece

**Keywords:** COVID-19, pandemic, outbreak, epidemic, Europe, infectious disease, non-communicable diseases, antimicrobial resistance, public health, vaccination, surveillance

## Abstract

Between 2020 and 2025, Europe has faced multiple interacting public health threats shaped by and following the COVID-19 pandemic. Alongside COVID-19, the region experienced other infectious disease events, including monkeypox, measles resurgence, legionellosis and acute hepatitis of unknown origin in children. At the same time, non-communicable disease burdens, including obesity, type II diabetes mellitus, disruption of chronic disease care, mental health disorders and increased problematic digital use, intensified during and after the pandemic period. Antimicrobial resistance (AMR) remained a major cross-cutting threat because it undermines the effective treatment of infections and weakens emergency preparedness. This narrative review synthesizes peer-reviewed articles and selected reports from international organizations for the 2020–2025 period, using COVID-19 as the organizing context for examining interconnected infectious, chronic and system-level threats. Across these topics, recurring themes included vaccination gaps, fragmented surveillance, disruption of routine care, health system inequities, misinformation and insufficient preparedness for cross-border threats. The review supports integrated surveillance, continuity plans for essential services, stronger vaccination and risk-communication strategies and sustained AMR stewardship within a One Health framework. Coordinated action across public health, primary care, mental health and chronic disease policy is essential for future resilience.

## 1. Introduction

Between 2020 and 2025, the European populations have been exposed to significant health risks caused by both infectious disease events and non-communicable disease burdens. This has led to a new and increasingly complex reality for public health in Europe, characterized by strong interconnectivity among nations and a growing need for health systems to become flexible and responsive to public health challenges. The COVID-19 pandemic in 2020 represented the most significant and alarming public health event in recent decades, generating high levels of morbidity and mortality in Europe [1]. The unequal distribution of access to healthcare during the pandemic demonstrated the structural problems existing between EU member states and individual European countries [2]. Additionally, the differences in preparedness among national health systems, as well as the differences in vaccination coverage rates, had a direct impact on levels of morbidity and mortality. These experiences underscored the need to examine recent public health threats not only as isolated episodes, but also as part of a broader landscape of system vulnerability and preparedness.

Although COVID-19 was one of the major public health events in Europe in recent years, it was certainly not the only health risk. The organizational failure of primary healthcare services and the delay in implementing vaccination programs led to the resurgence of diseases once believed to be under control, such as measles and polio [3]. Additionally, the emergence of other infectious diseases, such as monkeypox and legionellosis, highlighted the need to develop more robust and coordinated epidemiological surveillance and rapid response mechanisms.

At the same time, even more concerning, however, has been the rising trend of non-communicable diseases in Europe. The combination of declining prevention activities, delaying routine care and the increasing burden of lifestyle-related issues (e.g., sedentary lifestyles, poor diets, lack of physical activity) combined with the additional pressure on mental health caused by the pandemic, have all contributed to deterioration in mental health, obesity, type II diabetes mellitus and other chronic disorders [4,5]. These conditions substantially shaped population vulnerability, worsened clinical outcomes and increased long-term demand on health systems.

In response to what the European Union has learned from the COVID-19 pandemic, new structures have been established to enhance the preparedness and response to cross-border threats to public health in Europe. One such structure is HERA (European Health Emergency Preparedness and Response Authority) whose mission is to prevent, detect and quickly respond to health crises through the development and implementation of critical response measures such as vaccination programs [6,7,8]. However, the public health challenges of 2020–2025 have shown that institutional reforms alone are insufficient to address them unless they are accompanied by strong and stable political commitment and continuous investments in public health [9].

The aim of this narrative review is to synthesize how the COVID-19 pandemic interacted with other major public health threats in Europe from 2020 to 2025 by bringing together evidence on selected infectious disease events, non-communicable disease burdens and antimicrobial resistance (AMR). The review seeks to identify common patterns across these diverse topics, explain why they should be considered together from a preparedness perspective and derive concrete recommendations for surveillance, vaccination, continuity of care and policy coordination in Europe. Although this review is selective rather than exhaustive, the chosen examples were intended to capture transferable preparedness lessons across different modes of transmission and public health functions.

## 2. Materials and Methods

This paper was designed as a narrative review because its objective was to synthesize a broad and heterogenous body of evidence spanning selected infectious disease events, non-communicable disease burdens and antimicrobial resistance in Europe during the years 2020 to 2025, rather than answer a single narrowly defined question suitable for a systematic review or meta-analysis. Searches were conducted in the PubMed and Scopus databases using combinations of keywords and Boolean operators related to Europe, infectious disease events, non-communicable diseases, antimicrobial resistance, epidemiology, surveillance and public health. The search strategy included terms such as (“outbreak*” OR “pandemic*” OR “epidemic*” OR “antimicrobial resistance” OR “drug-resistant”) AND “Europe” AND (“infectious disease*” OR “communicable disease*” OR “COVID-19” OR “respiratory infection*” OR “viral outbreak*” OR “non-communicable disease*” OR “chronic disease*” OR “cardiovascular disease*” OR “diabetes” OR “mental health”) AND (“epidemiology” OR “surveillance” OR “public health” OR “incidence” OR “prevalence” OR “morbidity” OR “mortality” OR “disease burden”).

Eligible sources included peer-reviewed articles in English and selected reports from the World Health Organization (WHO), the European Centre for Disease Prevention and Control (ECDC) and the Organization for Economic Cooperation and Development (OECD) that addressed European populations and were published between 2020 and 2025. Titles and abstracts were screened for relevance, followed by full-text review when needed to determine whether a source contributed epidemiologic, surveillance, clinical or policy information aligned with the aim of the review. Gray literature (e.g., working papers; theses and dissertations; technical reports; conference proceedings that have not undergone a peer-review process; internal commercial reports) was excluded. Because this was a narrative review, formal risk-of-bias assessment and PRISMA-style study accounting were not undertaken; instead, the final synthesis was organized thematically into selected infectious disease events, non-communicable disease burdens, antimicrobial resistance and cross-cutting policy lessons.

## 3. Results

### 3.1. Infectious Diseases

#### 3.1.1. The COVID-19 Pandemic

COVID-19 represents the most significant global health crisis of the period and was caused by SARS-CoV-2. The initial waves of COVID-19 (2020–2021) followed by subsequent variant-driven surges, resulted in a high number of deaths among elderly populations and those with chronic conditions [10,11]. Although vaccines against COVID-19 were developed and deployed rapidly after their approval, mutations in the virus, including Delta and Omicron variants, created recurrent waves throughout Europe [12]. The COVID-19 pandemic demonstrated large disparities in how each European country responded to managing a public health crisis, and further increased the debate regarding whether all countries should act together in response to future pandemics [12]. The COVID-19 pandemic also generated profound social and economic disruption. Lockdowns, social distancing measures and teleworking created new daily routines for many individuals. It was also evident that there was widespread noncompliance by the general public with recommended prevention methods that negatively impacted the spread of COVID-19. Vaccines were one method used to slow down the spread of COVID-19; however, barriers to vaccination uptake existed including public concern regarding vaccine safety, public unwillingness to be vaccinated due to mistrust and inequitable early vaccine access, as well as misinformation, limited the effectiveness of vaccines in slowing down the spread of COVID-19 in some settings [13,14]. Overall, it is anticipated that the lasting impacts from COVID-19 will occur over a prolonged period of time, resulting in long-term implications for population health, economies and societies [15].

#### 3.1.2. Monkeypox (Mpox)

In 2022, a significant increase in monkeypox cases occurred in Europe, particularly among men having sex with men [16,17,18]. Compared with classical descriptions from endemic African settings, many European cases presented with genital or perianal lesions, proctitis, painful mucosal disease and other complications that broadened the recognized clinical spectrum [16,17,18]. The monkeypox epidemic did not reach the same level of incidence or mortality as that of COVID-19; however, it prompted the implementation of various public awareness mechanisms and recommendations for targeted vaccine campaigns. As part of these public awareness campaigns, there was a focus on promoting more responsible sexual behaviors [19]. The monkeypox epidemic served as a critical test for health authorities to determine their capacity to identify and respond to rare infectious diseases. This epidemic has clearly demonstrated the need for global cooperation in surveillance for zoonotic diseases, as well as information sharing between international and national health organizations.

#### 3.1.3. Measles and Poliovirus Detections

Measles requires separate attention because Europe had already experienced repeated outbreaks before the COVID-19 pandemic, often initiated by imported or import-related cases and then amplified by local immunity gaps [20]. The COVID-19 pandemic further disrupted routine immunization and catch-up activities, contributing to renewed transmission in several countries. Consequently, achieving optimal vaccination coverage has once again become a central priority for public health officials. Furthermore, the recent measles epidemic has highlighted the vulnerability of immunization programs during periods of crisis, particularly during the COVID-19 pandemic. Moreover, the growing distrust in vaccines as a result of the COVID-19 pandemic and subsequent misinformation campaigns may ultimately threaten decades of progress made toward the control of measles.

Poliomyelitis presents a different epidemiological pattern. Although Europe remained free of endemic paralytic polio, wastewater surveillance in 2024 detected vaccine-derived poliovirus type 2 (VDPV2) in Finland, Germany, Poland, Spain and the United Kingdom, highlighting the continuing risk of silent circulation when immunity gaps persist [21]. This reinforced the importance of high vaccination coverage, environmental surveillance and rapid cross-border risk assessment.

#### 3.1.4. Legionellosis

In June 2023, a Legionnaires’ disease outbreak was reported in Poland; the majority of these cases were located near the city of Rzeszow, with over 160 total cases of Legionnaires’ disease and 19 associated deaths among the elderly or immunocompromised people [22]. The Polish authorities were able to identify that the most likely source of the outbreak was the community water supply system. Because Legionnaires’ disease is caused by Legionella pneumophila and transmitted through inhalation of contaminated water droplets, this outbreak has demonstrated the importance of routinely inspecting water quality as well as being aware of less common but severe infections such as Legionnaires’ disease.

#### 3.1.5. Acute Hepatitis of Unknown Origin in Children

Global concerns were documented in early 2022 due to an increase in reported cases of unexplained acute hepatitis among children. A substantial number of cases were documented throughout Europe during this period [23,24]. Patients displayed clinical manifestations consistent with typical acute hepatitis (such as jaundice, vomiting and abdominal pain), but no hepatitis viruses could be identified through testing. Several of these cases resulted in severe disease, which required liver transplant. Scientific hypotheses regarding the causes of this syndrome included potential association with adenovirus infection or an abnormal immune response to previous SARS-CoV-2 infection [25]. This has clearly demonstrated that there is a need for prompt and effective surveillance for unusual syndromes and coordinated investigation of uncertain etiologies.

Table 1 summarizes the major infectious disease events discussed in this review.

### 3.2. Non-Communicable Diseases

#### 3.2.1. Mental Health

The COVID-19 pandemic was associated with a substantial deterioration in mental health among European populations [26,27]. Rapidly increasing rates of depression and anxiety have developed particularly among children, young adults and healthcare workers [28,29]. There was also inadequate preparation of mental health services to meet the high level of demand created by the pandemic, which resulted in delays in access to care and unmet healthcare needs [30]. Depressive symptoms, suicide attempts, and, in some settings, suicides also increased or became a major public health concern in the pandemic and post-pandemic context [31,32,33]. Notably, particular attention should be given to the impact of the pandemic on children and adolescents, as increases in anxiety, sleep disturbances and eating disorders have already occurred [34].

#### 3.2.2. Obesity

Pandemic restrictive measures promoted sedentary behavior and led to a worsening of obesity indicators, especially among children and adolescents [35,36]. Obesity is a significant risk factor for many chronic health conditions including type II diabetes mellitus and cardiovascular diseases. Furthermore, changes in lifestyles, psychological stress and prolonged confinement have negatively impacted an individual’s dietary quality [37,38,39]. Changes in eating behaviors and an increase in consumption of processed foods during the lockdown have been reported [40,41]. Vulnerable populations experienced additional challenges when accessing healthy food options was further restricted [42,43].

#### 3.2.3. Chronic Diseases

The reallocation of healthcare resources toward the management of COVID-19 significantly disrupted routine care for chronic diseases, including cardiovascular and respiratory conditions [44,45]. Delayed appointments, deferred treatment and interruptions in monitoring have led to poorer disease control and an increase in preventable deaths [10,46]. The suspension of preventative screening reduced the opportunity for early diagnosis and timely intervention [47]. Emerging evidence also suggests that SARS-CoV-2 infection may contribute to cardiovascular complications, particularly among younger populations, further increasing the burden on healthcare systems [48].

#### 3.2.4. Type II Diabetes Mellitus

Type II diabetes mellitus has been identified as one of the fastest-growing chronic diseases in Europe. During the pandemic period, reduced physical activity, unhealthy dietary patterns, and disruptions in routine healthcare services contributed to delayed diagnosis and weaker disease control [49,50]. Type II diabetes mellitus significantly increases the risk of serious complications, including cardiovascular disease, renal failure and neuropathy. Effective diabetes management relies on four key components: targeted preventive strategies, educational interventions for patients, utilizing digital technologies to monitor blood glucose levels on a regular basis and providing all individuals with equitable access to primary care services [51,52]. Addressing the social determinants associated with the development of type II diabetes should remain part of both national and European prevention programs.

#### 3.2.5. Digital Addiction

In recent years, dependence on digital platforms for education, work, interaction, and entertainment has increased substantially. This has resulted in greater screen time among adolescents, which has been linked to disrupted sleep patterns, reduced physical activity levels, concentration difficulties, and declining mental health [53,54]. The lack of opportunity for in-person socialization combined with being constantly exposed to the many types of digital stimuli, including video games and social media, contributes to an increased risk of developing an addiction [55,56].

### 3.3. Antimicrobial Resistance

During 2020–2025, concern about AMR in Europe intensified because of antibiotic pressure, persistent healthcare-associated transmission and uneven infection-prevention capacity, which threatened to expand the burden of multidrug-resistant infections. Although AMR does not resemble an acute point-source epidemic, it spreads across populations and healthcare systems, undermines the treatment of common infections and can amplify the impact of future outbreaks by reducing therapeutic options. Therefore, AMR is viewed as a public health threat and is often referred to as a “silent epidemic” or “ongoing pandemic”. It is also worth noting that AMR is identified as one of the top 10 threats to global health by the WHO and other organizations [57]. Implementing surveillance systems, providing rational antimicrobial stewardship and developing new therapeutic and diagnostic tools are key interventions [58,59]. Additionally, addressing AMR through a coordinated, One Health-type approach that considers human, animal and environmental health is essential [60,61,62].

Table 2 summarizes the major non-communicable disease and cross-cutting public health burdens discussed in this review.

## 4. Discussion

The findings of this narrative review suggest that the public health threats of 2020–2025 in Europe should not be interpreted as isolated episodes. Viewed through the organizing lens of COVID-19, the selected infectious disease events (mpox, measles resurgence, poliovirus detections, legionellosis and pediatric hepatitis), together with the disruption of routine care for chronic diseases, mental health deterioration and AMR, exposed recurring health system weaknesses: gaps in surveillance, vulnerability of routine vaccination programs, delayed communication, fragmentation between countries and insufficient protection of essential health services during crises [18,19,20,21,22,23,24,25,44,45,46,47,48].

Vaccination emerged as one of the clearest connecting themes. The infectious disease events reviewed here repeatedly exposed the consequences of declining routine immunization coverage and vaccine hesitancy, while the COVID-19 experience showed that rapid vaccine development alone is not enough when access is unequal and public trust is weak [13,14,20,21]. From a preparedness perspective, vaccination policy in Europe must therefore address both delivery capacity and community confidence, including catch-up programs for measles and polio and targeted vaccination strategies for emerging threats such as mpox [19,20,21].

A second recurring pattern was the interaction between infectious crises and non-communicable disease burden. Chronic diseases and obesity increased vulnerability to severe infectious outcomes, while service disruption and behavioral changes during the pandemic worsened mental health, diabetes and long-term disease control [26,27,28,29,30,35,36,37,38,39,40,41,42,43,44,45,46,47,48,49,50,51,52]. Preparedness cannot therefore be limited to outbreak containment alone; it must also include continuity plans for primary care, mental health services, screening and chronic disease follow-up.

Finally, this review has limitations: it is a narrative review rather than a systematic review and it therefore does not provide exhaustive study capture, formal risk-of-bias assessment, or quantitative synthesis. However, this broader approach was appropriate for integrating infectious disease events, non-communicable disease burdens and system-level lessons across Europe within a single interpretive framework.

## 5. Policy Implications and Recommendations

As the European Union has clearly demonstrated through its efforts to counteract the COVID-19 pandemic by strengthening institutions like the ECDC and establishing HERA to improve the EU’s preparedness and response to future pandemics [63], the need for a unified strategy for managing health risks and for investment in both disease prevention and health promotion remains urgently important [64]. Any cooperation among member states to manage chronic diseases and mental health issues are essential components of this process.

First, Europe should protect routine immunization and implement catch-up vaccination strategies as a standing preparedness priority. This recommendation follows directly from the COVID-19 vaccination challenges and from the resurgence of measles described in this review [13,14,20,21]. Vaccination strategies should combine delivery capacity, targeted outreach to under-immunized groups and communication approaches that address misinformation and rebuild trust. Second, surveillance systems should be strengthened across clinical, laboratory, environmental and cross-border platforms. The reviewed events showed the value of rapid data sharing for zoonotic threats and unusual syndromes, while legionellosis and acute hepatitis in children illustrated the importance of environmental and syndrome-based surveillance [22,23,24,25]. Greater interoperability, earlier notification and stronger coordination through European institutions remain essential.

Third, preparedness plans must explicitly protect continuity of care for chronic diseases and mental health during emergencies. Primary care, preventive screening, diabetes follow-up and mental health services should not be treated as secondary functions that can be suspended without mitigation plans [26,27,28,29,30,44,45,46,47,48,49,50,51,52]. Flexible service delivery models, including telemedicine and targeted outreach, should be linked to equity-focused policies for vulnerable populations. Fourth, AMR should be treated as a standing preparedness priority rather than as a separate technical agenda. Europe needs sustained antimicrobial stewardship, infection prevention and control, improved diagnostics and One Health surveillance across human, animal and environmental domains [57,58,59,60,61,62]. Last but not least, integrating health considerations across all policy sectors could significantly enhance the European Union’s capacity to promote and protect the health and well-being of its populations. The Health in All Policies (HiAP) approach is a contemporary public health strategy that focuses on integrating health considerations into all policy sectors [65]. HiAP recognizes that social, environmental and economic determinants play a critical role in shaping population health, and therefore seeks to develop intersectoral collaboration among government agencies to improve health indicators and reduce inequities in health outcomes.

## 6. Conclusions

Between 2020 and 2025, Europe faced a combination of COVID-19, other infectious disease events, non-communicable disease burdens and AMR that exposed shared weaknesses in preparedness, vaccination delivery, surveillance, communication and continuity of care. This has highlighted the need for an integrated and sustainable public health approach to address and prevent future pandemics through the development and implementation of effective strategies. To develop these strategies and to build upon the foundation of prevention, research and population education, there is a critical need to strengthen the epidemiologic capacity of European health systems to effectively adapt to rapidly evolving conditions. Additionally, the growing burden of non-communicable diseases and the silent epidemic of AMR require sustained long-term prevention and health promotion efforts. A resilient European response therefore requires not only emergency structures, but also sustained investment in routine immunization, primary care, mental health, chronic disease management, antimicrobial stewardship and cross-border data systems. Future public health policy should be judged by its ability to protect essential services during crises while reducing inequities in access to prevention and care. Finally, the lessons learned from the experiences of recent years may provide an important foundation for revising public health strategies not only to manage future crisis situations, but also to promote resilience and sustainability of the health systems of European countries and protect their citizens.

## Figures and Tables

**Table 1:** Major infectious disease outbreaks discussed in the review.

Outbreak	Main European Pattern	Public Health Implications
COVID-19 (2020–2025)	Dominant health crisis of the period; early waves caused high mortality, while Delta and Omicron renewed transmission and pressure on health systems.	Excess mortality, social and economic disruption and a need for coordinated preparedness, communication and equitable vaccination.
Mpox (2022)	Rapid rise in cases in several Western European countries and concentration among men who have sex with men.	Called for targeted surveillance, community engagement, non-stigmatizing communication and focused vaccination.
Measles resurgence/poliovirus detection	Measles resurgence linked to falling vaccination coverage, pandemic-related service disruption and misinformation, especially in Eastern and South-Eastern Europe, while 2024 VDPV2 wastewater detections in five countries highlighted continuing polio risk.	Made catch-up immunization and restored confidence in routine vaccination urgent.
Legionellosis (Poland, 2023)	Cluster centered on Rzeszow and nearby areas, likely associated with the water supply system and affecting mainly older or immunocompromised people.	Highlighted the need for water-quality monitoring, environmental investigation and rapid local response.
Acute hepatitis of unknown origin in children (2022)	Europe recorded notable pediatric cases; some were severe and required liver transplantation, while the cause remained uncertain.	Strengthened the case for rapid syndrome-based surveillance and coordinated etiologic investigation.

**Table 2:** Major non-infectious and cross-cutting health burdens discussed in the review.

Health Burden	Main Drivers/Pattern	Public Health Implications
Mental health	Isolation, uncertainty, service disruption and prolonged stress raised depression and anxiety, suicidal behavior and other mental health problems, especially among children, adolescents and health workers.	Longer waiting times, unmet need and concern about suicide trends.
Obesity	Sedentary behavior, poorer diet, processed food intake and food insecurity worsened weight-related risk, especially in children and adolescents.	Higher long-term burden of cardiovascular disease, diabetes and other chronic conditions.
Chronic diseases	COVID-19 redirected resources, delaying routine visits, screening and treatment for cardiovascular, respiratory and other chronic conditions.	Worse disease control, preventable deaths and later diagnosis.
Type II diabetes mellitus	Lower activity, poorer diet and delayed monitoring led to late diagnosis and weaker glycemic control.	Greater cardiovascular, renal and neurologic complication risk; stronger primary care follow-up is needed.
Digital addiction	Online schooling and digital social life increased screen exposure, particularly among adolescents.	Sleep problems, lower physical activity, concentration difficulties and poorer mental health.
Antimicrobial resistance	Antibiotic overuse and weak infection prevention sustained the spread of resistant microorganisms in human and animal settings.	A persistent “silent epidemic” needing stewardship, surveillance and a One Health response.

## Data Availability

No new data were created or analyzed in this study. Data sharing is not applicable to this article.
